# A novel compression garment with adhesive silicone stripes improves repeated sprint performance – a multi-experimental approach on the underlying mechanisms

**DOI:** 10.1186/2052-1847-6-21

**Published:** 2014-05-30

**Authors:** Dennis-Peter Born, Hans-Christer Holmberg, Florian Goernert, Billy Sperlich

**Affiliations:** 1 Department of Sport Science, University of Wuppertal, Fuhlrottstraße 10, 42119 Wuppertal, Germany; 2 Swedish Winter Sports Research Centre, Department of Health Sciences, Mid Sweden University, 83125 Östersund, Sweden; 3 Department of Sport Sciences, Integrative and Experimental Exercise Science, University of Würzburg, 97082 Würzburg, Germany

**Keywords:** Blood flow, Clothing, Oscillation, Oxygenation, Oxygen uptake, Textile, Tissue saturation index, Venous system, Video analysis

## Abstract

**Background:**

Repeated sprint performance is determined by explosive production of power, as well as rapid recovery between successive sprints, and there is evidence that compression garments and sports taping can improve both of these factors.

**Methods:**

In each of two sub-studies, female athletes performed two sets of 30 30-m sprints (one sprint per minute), one set wearing compression garment with adhesive silicone stripes (CGSS) intended to mimic taping and the other with normal clothing, in randomized order. Sub-study 1 (n = 12) focused on cardio-respiratory, metabolic, hemodynamic and perceptual responses, while neuronal and biomechanical parameters were examined in sub-study 2 (n = 12).

**Results:**

In both sub-studies the CGSS improved repeated sprint performance during the final 10 sprints (best *P* < 0.01, *d* = 0.61). None of the cardio-respiratory or metabolic variables monitored were altered by wearing this garment (best *P* = 0.06, *d* = 0.71). Also during the final 10 sprints, rating of perceived exertion by the upper leg muscles was reduced (*P* = 0.01, *d* = 1.1), step length increased (*P* = 0.01, *d* = 0.91) and activation of the *m. rectus femoris* elevated (*P* = 0.01, *d* = 1.24), while the hip flexion angle was lowered throughout the protocol (best *P* < 0.01, *d* = 2.28) and step frequency (best *P* = 0.34, *d* = 0.2) remained unaltered.

**Conclusion:**

Although the physiological parameters monitored were unchanged, the CGSS appears to improve performance during 30 30-m repeated sprints by reducing perceived exertion and altering running technique.

## Background

Performance during repeated sprints separated by short periods of rest, a common aspect of team sports [1,2], is determined primarily by neuronal and metabolic factors (e.g., recruitment of muscle fibers and aerobic and anaerobic energy production [3,4]), as well as by the ability to recover between repeated bouts of explosive high-intensity activity [4,5]. Special running gear might improve this performance and, indeed, a recent meta-analysis revealed that lower-body garments that exert compression probably have ergogenic effects during sprinting [6]. Although the mechanism (s) underlying the improvement of repeated sprint performance by compression is not yet clear, the enhanced muscle pump function and venous return [7,8] observed in clinical studies have been proposed as explanation for the elevated local hemodynamics during moderate [9], as well as intermittent high-intensity running [10]. Moreover, reduced blood lactate concentrations following high [11,12] or reduced oxygen uptake during moderate-intensity endurance exercise [13] were also associated with an ergogenic effect of compression clothing on local and central hemodynamics.

In a manner similar to compression garments, application of elastic tape, originally used for therapeutic purposes [14], has been found to improve strength and power [15,16], although a recent meta-analysis was inconclusive with respect to muscle activity and pain [17]. Taping is thought to stimulate cutaneous mechano- and nocireceptors, thereby altering reflex activation and sensory feedback and increasing electromyographic activity [18,19], thereby enhancing proprioception [20] and muscle strength and power [15,16].

Since compression and taping each influence physiological, biomechanical and perceptual parameters, the primary goal of the present investigation was to examine whether the use of both together improves repeated sprint performance even further. In this context we tested a novel compression garment with adhesive silicone stripes (CGSS) designed to mimic both compression and sports taping. Our secondary goal was to identify the physiological, biomechanical and perceptual effects that might account for the ergogenic properties of this garment.

## Methods

To reduce the necessity for bulky laboratory equipment during repeated sprinting, two sub-studies were performed: The first to investigate the influence of CGSS on repeated sprint performance and cardio-respiratory, metabolic, hemodynamic and perceptual variables; and the second to examine whether this special garment might exert an impact on biomechanical properties and running technique.

A total of 24 female subjects were recruited from track-and-field or team sport clubs that regularly use repeated sprinting as part of their training and randomly assigned to participate in sub-study 1 or 2. These women were instructed to report for all testing well-hydrated and to refrain from strenuous exercise for 48 h and from intake of alcohol or caffeine for 24 h beforehand. After being informed of the benefits and risks involved, all provided their written consent to participate. This study was approved by the Ethical Committee of the University of Wuppertal, Germany, and executed in accordance with the Declaration of Helsinki.

### Design of sub-study 1

All athletes in sub-study 1 (n = 12; age: 25 ± 3 yrs; height: 167 ± 3 cm; body mass: 61 ± 5 kg; fat mass: 18 ± 5% (mean ± SD)) carried out two sessions of 30 30-m repeated sprints each, one wearing the CGSS and the other with non-compression tights without any adhesive silicone stripes, in randomized order. Each participant wore the same shoes and running shirt during both trials. All sprints were performed on an indoor track at a time when the subjects were not menstruating.

**Figure 1:**
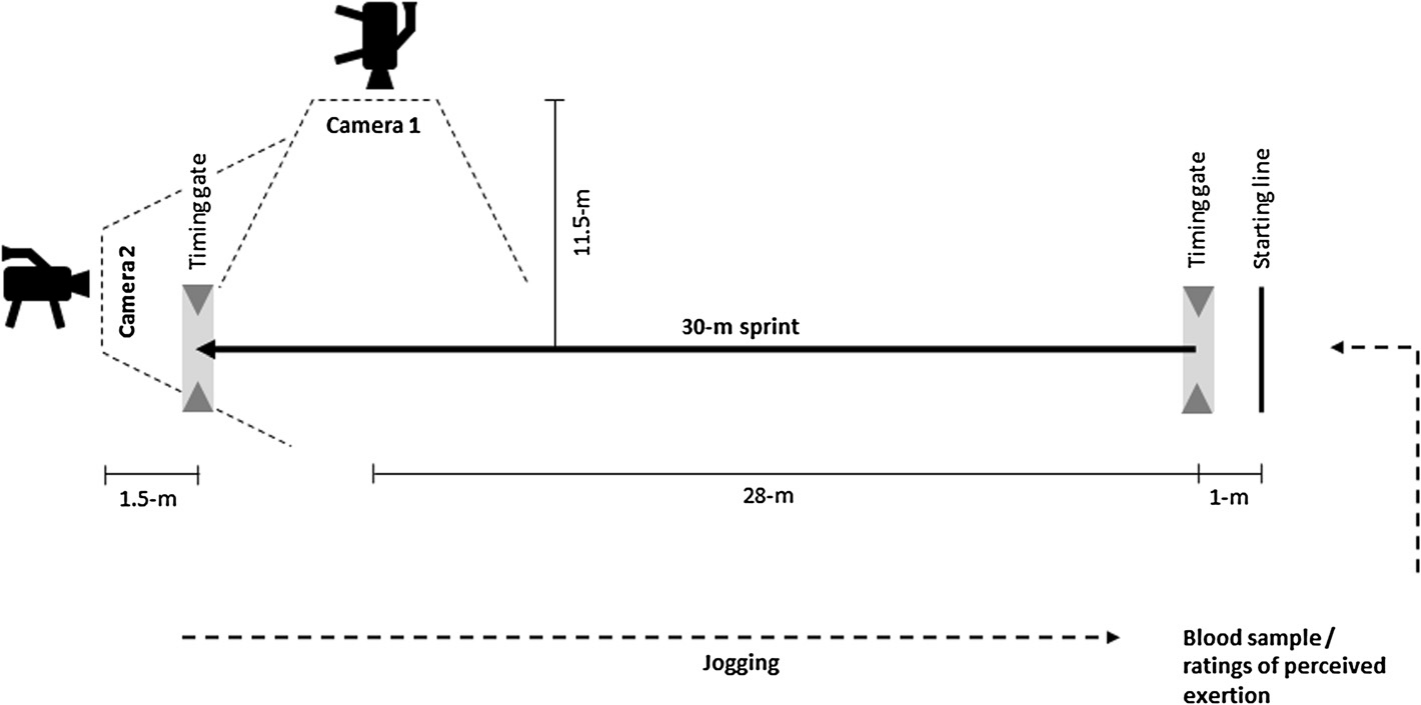
Schematic illustration of the repeated sprint protocol.

After determination of body mass (Tanita BC 418 MA, Tanita Corp., Tokyo, Japan), the athletes warmed up for 20 min with moderate running, including five 5-m and 10-m sprints. During the repeated sprint protocol, each sprint was initiated by a verbal count-down once each minute. Starting 1 m behind the first gate, they were instructed to complete each 30-m sprint as fast as possible, avoiding pacing, and thereafter to jog back to the starting line at a moderate pace. No feedback concerning performance was provided, in order to promote equal motivation to complete each sprint. Sprint times were recorded by timing gates (TDS Werthner Sport Consulting, Linz, Austria) at the start and end of the 30-m track.During these trials the women wore a portable telemetric metabolic cart, a chest belt that monitored heart rate, and a portable near-infrared spectroscope (NIRS). Blood samples and ratings of perceived exertion were obtained at the same point in each cycle of sprinting (Figure 1).

### Design of sub-study 2

All athletes in sub-study 2 (n = 12; age: 23 ± 2 yrs; height: 169 ± 3 cm; body mass: 61 ± 6 kg; fat mass: 17 ± 4% (mean ± SD)) carried out the same procedure as in sub-study 1, but wore instead a telemetric device to record muscle activation during the repeated sprinting. In this case motion was captured by video-analysis.

### The compression garment with adhesive silicone stripes

**Figure 2:**
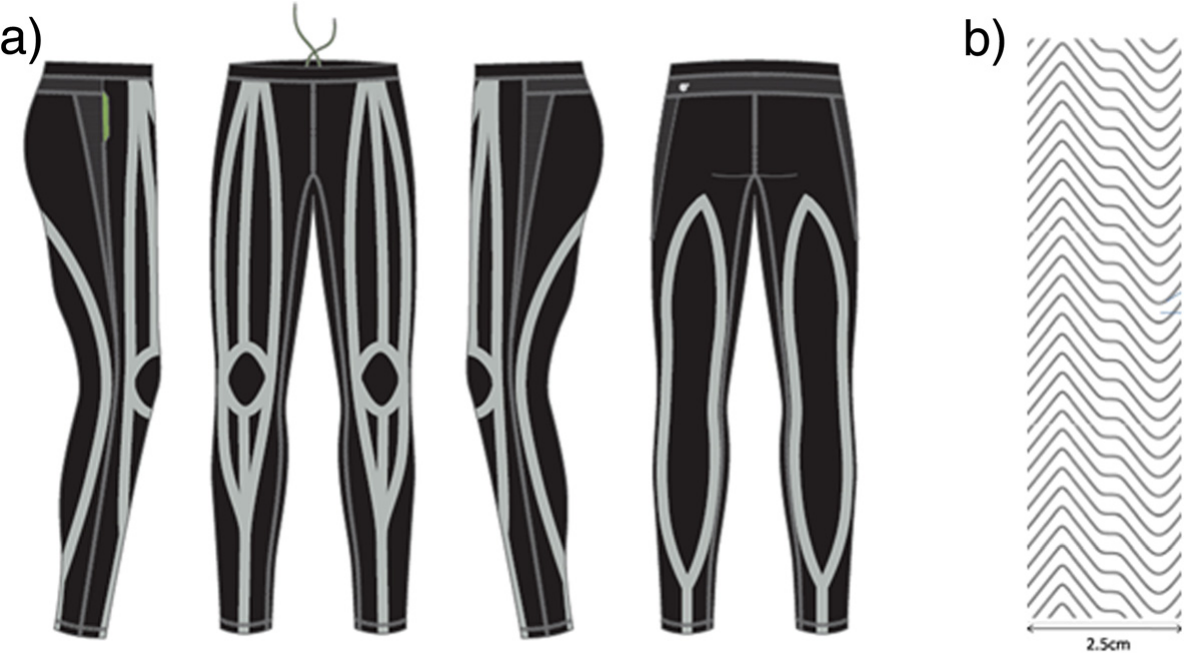
Schematic illustration of the CGSS with placement of the silicone stripes on the anterior and posterior leg (a) and detailed structure of the adhesive silicone stripes on the inner lining of the garment (b).

The CGSS consisted of tights extending from the waist to the ankle with adhesive stripes of silicone on the inner lining designed to compress the anterior and posterior thighs, as well as lower legs, in accordance with previous guidelines [21] (Figure 2). Before the sprints, the levels of compression on the skin by this garment at the *m. gluteus maximus, m. rectus femoris, m. vastus lateralis, m. biceps femoris* and *m. gastrocnemius medialis* were determined three times in accordance with international recommendations employing a pneumatic sensor (SIGaT®, Ganzoni-Sigvaris, St. Gallen, Switzerland) [22], as described previously [23,24].

### Data collection

In sub-study 1 gas exchange and heart rate were monitored continuously with a portable breath-by-breath gas analyzer (Metamax 3B, Cortex, Leipzig, Germany) and a chest belt (Polar T31, 1 Hz, Polar Electro Oy, Kempele, Finland), respectively. The gas analyzer used standard algorithms to account for the time delay between inspiration and expiration and to calculate the oxygen consumption. The volume was calibrated using a syringe with a volume of exactly 3 L (Cortex, Leipzig, Germany). The anticipated range of fractional gas concentrations was calibrated before each trial with 15.8% O_2_ and 5% CO_2_ in N_2_ (Praxiar Technology Inc., Danbury, CT, USA). A Hans-Rudolph mask was used to attach the turbine flowmeter and all tubes and cables were fixed in place with tape and straps to assure that the athletes could move freely.

For determination of alternations in muscle oxygenation and blood volume, a wireless NIRS (Portamon, Artinis Medical System, Zetten, The Netherlands) was attached with adhesive tape between the lateral femoral epicondyle and the greater trochanter of the femur across the maximum girth of the belly of the right *vastus lateralis* muscle. None of the participants reported discomfort or disturbance of their normal pattern of movement by any of the devices attached. Since the pattern of oxygenation within the *vastus lateralis* muscle is not uniform, the location of the NIRS during the first trial was indicated with a permanent marker to ensure the same placement during the second set of 30 30-m sprints [25].

The NIRS weighed 85 g and measured 83 × 52 × 20 mm, with distances of 30, 35 and 40 mm between the three light sources and the optical detector. Changes in the oxygenated and deoxygenated levels of hemoglobin (Hb) plus myoglobin (Mb) were monitored at 760 and 890 nm, respectively [26]. In addition, local blood volume was determined on the basis of total hemoglobin (tHb), as indicated by the sum of these signals. The tissue saturation index [%] (TSI%), reflecting the relationship between oxygen delivery and consumption, was calculated as [[HbO_2_]/([HbO_2_] + [HHB)] × 100.

At the same point in each cycle of sprinting, capillary blood was sampled from the right earlobe for determination of the lactate concentration by amperometric-enzymatic analysis (Ebio Plus, Eppendorf AG, Hamburg, Germany) and subjective ratings of perceived exertion by the whole body, upper leg muscles (including the gluteal muscles) and lower leg muscles were made on Borg’s 6 – 20 scale [27]. Skin temperature on the anterior thigh was determined (MSR Modular Signal Recorder, Prospective Concepts AG, Glattbrugg, Switzerland) both before and after each set of sprints to ensure equal thermoregulatory conditions for both sets.

In sub-study 2 each sprint was recorded with a high-speed camera at a sampling rate of 120 Hz (GoPro, San Mateo, CA, USA) and the kinematic data collected analyzed digitally (Kinovea®, version 0.8.15, Bordeaux, France). This camera was placed 11.5 m away from and at a right angle to a point 28 m along the running track (Figure 1). Markers were attached to the athlete’s greater trochanter, medial and lateral femoral epicondyles, as well as to both the medial and lateral forefoot. As previously, markers were fixed to the garment in order not to interfere with its compression properties [28]. To determine step frequency, a second camera was positioned at the end of the running track, 1.5 m to the side of the finish gate to record each step. The number of steps taken during each sprint was counted and divided by the sprint time to obtain step frequency, as described previously [29].

Surface electromyographic activity (EMG) was measured on the right side of the body utilizing pre-gelled bipolar AgCl electrodes (Blue Sensor N, Ambu A/S, Ballerup, Denmark). After shaving, abrading, and disinfecting the skin, these electrodes were attached parallel to the muscle fibers on the skin of the bellies of the *gluteus maximus, rectus femoris, vastus lateralis, biceps femoris,* and *gastrocnemius medialis* muscles. A reference electrode was attached to the tibial bone and all positioning was as recommended by Hermens and Hermie [30]. All signals were transferred to the computer by an analog-to-digital converter card (DAQ 700 A/D card, National Instruments, Austin, TX, USA), differentially amplified and stored in real time by a telemetric recording system (EMG, TeleMyo2400T, Noraxon Inc., Scottdale, AZ, USA). The sampling rate was 1500 Hz. To erase low- and high-frequency noise, all raw signals were passed through a digital band-pass filter (10-500 Hz, 3 dB, Butterworth 2^nd^ edition). The signals were then rectified, smoothed (using the mean values for 50-ms time frames), and normalized to the fastest sprint, as recommended earlier [31]. The integrated EMG (iEMG) was employed as an indicator of muscle activation. All of these analyses were performed with the MyoResearch program (Master Edition version 1.08.27, Noraxon Inc., Scottsdale, AZ, USA).

### Statistical analysis

All data are presented as mean values (mean) ± standard deviations (SD). As decided in advance, we were interested in comparing the CGSS and control conditions. After confirming normal distribution, the data from each set of repeated sprints were divided into three equal subsets (sprints 1-10, 11-20, and 21-30), these subsets averaged and data for CGSS and control condition compared using Student’s paired *t*-test. A difference with an alpha value of *P* ≤ 0.05 was considered to be statistically significant and then adjusted for multiple comparison with the conservative Bonferroni correction, as described previously [32-34]. All of these analyses were performed with the Statistica software package for Windows (version 7.1, StatSoft Inc., Tulsa, OK, USA).

To compare the practical relevance and meaningfulness of the various findings, effect sizes were calculated using the conventional procedure proposed by Cohen [35]. Cohen’s *d* value was calculated by dividing the difference between the means of the intervention and control trials by the average standard deviation for the subject population [35]. In the conventional manner, effect sizes of 0.20, 0.50 and 0.80 were regarded as small, medium and large, respectively [35].

## Results

Table 1 documents the pressure applied on the skin by the garment at the various sites on the body during both sub-studies. All physiological, biomechanical and performance values, as well as the level of statistical significance (*P*) and corresponding effect size (*d*) for all comparisons made, are presented in Table 2.

**Table 1:** The pressure exerted at various sites on the skin by the compression garment with adhesive silicone stripes

**Muscle**	**Pressure [mmHg] (mean ± SD)**	
	**Sub-study 1 (n = 12)**	**Sub-study 2 (n = 12)**
*Gluteus maximus*	18.3 ± 4.1	20.2 ± 4.3
*Rectus femoris*	19.0 ± 4.9	20.2 ± 4.9
*Vastus lateralis*	17.5 ± 4.4	18.2 ± 4.1
*Biceps femoris*	19.6 ± 4.7	19.5 ± 5.6
*Gastrocnemus medialis*	21.7 ± 6.0	19.9 ± 5.6

**Table 2:** Physiological, biomechanical and performance values (means ± SD) associated with the repeated sprints with and without the compression garment with adhesive silicone stripes (CGSS)

**Variable**	**Sprints 01-10**				**Sprints 11-20**				**Sprints 21-30**			
	**CGSS**	**Control**	***P *** **value**	**ES *d***	**CGSS**	**Control**	**P value**	**ES *d***	**CGSS**	**Control**	**P value**	**ES *d***
**Sub-study 1 (n = 12)**												
Time [s]	4.97 ± 0.21	5.00 ± 0.22	0.28	0.22	5.09 ± 0.29	5.12 ± 0.29	0.09	0.14	5.12 ± 0.29	5.21 ± 0.34	0.02	0.37
Oxygen uptake [mL∙min^-1^]	2.51 ± 0.53	2.45 ± 0.48	0.47	0.17	2.62 ± 0.63	2.65 ± 0.64	0.77	0.08	2.59 ± 0.6	2.63 ± 0.64	0.71	0.09
Ventilation [L∙min^-1^]	69.1 ± 9.9	68.3 ± 12.6	0.68	0.1	76.5 ± 7.1	78 ± 10.5	0.53	0.23	77.3 ± 8.3	79.1 ± 9.1	0.3	0.31
Heart rate [bpm]	169 ± 7.5	169 ± 7.5	0.92	0.04	178 ± 8.2	177 ± 5.5	0.59	0.2	177 ± 8.5	178 ± 7.2	0.45	0.2
Blood lactate [mmol∙L^-1^]	5.53 ± 1.54	6.17 ± 2.03	0.13	0.5	6.55 ± 1.92	7.29 ± 1.9	0.16	0.54	6.46 ± 2.03	6.84 ± 1.74	0.45	0.29
Tissue saturation index [%]	93.3 ± 3	92.9 ± 3.9	0.68	0.14	91.2 ± 3.4	92.2 ± 4	0.27	0.35	90.7 ± 3.5	91.5 ± 4.6	0.34	0.27
Oxy-hemoglobin [μM∙cm]	101 ± 10.8	95 ± 10.4	0.06	0.71	108 ± 14.5	107 ± 17.2	0.8	0.08	109 ± 15.7	110 ± 22.6	0.83	0.07
Deoxy-hemoglobin [μM∙cm]	108 ± 6.3	108 ± 7.7	0.87	0.06	113 ± 7.7	116 ± 11.7	0.26	0.42	114 ± 8.7	119 ± 16.9	0.25	0.51
Total hemoglobin [μM∙cm]	103 ± 7.2	100 ± 8	0.4	0.44	109 ± 8.7	111 ± 14.4	0.61	0.21	110 ± 9.5	113 ± 20	0.41	0.33
Rating of perceived exertion [Borg’s Scale]												
Whole-body	10 ± 2.3	10 ± 2.3	0.98	0.01	14.6 ± 2.2	15.4 ± 1.9	0.09	0.58	17.6 ± 2.3	18.5 ± 1.3	0.13	0.68
Upper leg muscles	9.5 ± 2.7	10.4 ± 2.5	0.29	0.49	13 ± 3.3	14.9 ± 1.7	0.04	1.0	15.7 ± 3.2	17.7 ± 1.9	0.01	1.1
Lower leg muscles	8.6 ± 2.3	8.3 ± 2.3	0.5	0.19	11.4 ± 3.2	11.7 ± 3.2	0.58	0.12	13.6 ± 3.7	14 ± 4	0.64	0.15
**Sub-study 2 (n = 12)**												
Time [s]	4.87 ± 0.31	4.88 ± 0.39	0.73	0.07	4.88 ± 0.31	4.95 ± 0.40	0.08	0.25	4.85 ± 0.31	4.99 ± 0.36	<0.01	0.61
Hip flexion angle [°]	95.3 ± 4.6	102.8 ± 7.5	<0.01	1.69	96.4 ± 6.3	106.7 ± 6.4	<0.01	2.28	98.4 ± 6.4	106.3 ± 6.1	<0.01	1.78
Step length [m]	2.24 ± 0.1	2.17 ± 0.13	0.03	0.85	2.23 ± 0.13	2.16 ± 0.16	0.07	0.61	2.24 ± 0.14	2.14 ± 0.17	0.01	0.91
Step frequency [Hz]	3.66 ± 0.23	3.65 ± 0.21	0.78	0.08	3.64 ± 0.23	3.65 ± 0.26	0.91	0.03	3.66 ± 0.24	3.63 ± 0.26	0.34	0.2
iEMG [%]												
*Gluteus maximus*	91.8 ± 11.8	81.7 ± 13.3	0.04	1.14	91.5 ± 28.3	83.5 ± 19.6	0.37	0.46	93.9 ± 31.9	79.2 ± 14	0.13	0.84
*Rectus femoris*	92.7 ± 12.6	96.7 ± 13.5	0.36	0.44	91.1 ± 11	86.8 ± 15.5	0.5	0.45	95.4 ± 18.5	78.9 ± 19.2	0.01	1.24
*Vastus lateralis*	88.6 ± 14.7	84.2 ± 6.5	0.31	0.55	89.8 ± 19.9	85.1 ± 13.1	0.53	0.39	95.1 ± 23.9	84.7 ± 10.6	0.23	0.8
*Biceps femoris*	91.3 ± 21.8	90.3 ± 6.3	0.88	0.08	87.7 ± 19.3	85.8 ± 13.8	0.79	0.16	92.1 ± 24.4	83.8 ± 15.5	0.32	0.58
*Gastrocnemius medialis*	88.8 ± 18.7	86.5 ± 13.4	0.63	0.21	92 ± 28.7	89.8 ± 15.5	0.79	0.13	96.2 ± 39.5	102.4 ± 24.5	0.65	0.26

Abbreviations. iEMG, integrated EMG; ES, Effect size.

### Sub-study 1: Cardio-respiratory, metabolic, hemodynamic and perceptional variables during repeated sprinting

Performance of the repeated 30 30-m sprints was improved during the final third of the protocol (sprints 21-30) by wearing the CGSS (*P* = 0.02, *d* = 0.37). Cardio-respiratory and metabolic values (best *P* = 0.13, best *d* = 0.54), the tissue saturation index (best *P* = 0.27, *d* = 0.35), levels of oxy- (best *P* = 0.06, *d* = 0.71), deoxy- (best *P* = 0.25, *d* = 0.51) and total hemoglobin (best *P* = 0.4, *d* = 0.44) as well as skin temperature (*P* = 0.75, *d* = 0.18) were all unaffected by the use of this garment. Furthermore, during the final 10 sprints the CGSS reduced the perceived rating of exertion in the upper leg muscles (*P* = 0.01, *d* = 1.1), but not in the lower leg muscles (*P* = 0.64, *d* = 0.15) or for the whole body (*P* = 0.13, *d* = 0.68).

### Sub-study 2: Biomechanical parameters during repeated sprinting

In sub-study 2, the repeated 30-m sprint times were again significantly improved during the final third of the protocol by wearing the CGSS (*P* < 0.01, *d* = 0.61). Motion capture analysis revealed that this garment significantly reduced the hip flexion angle (best *P* < 0.01, *d* = 2.28). Moreover, during the final 10 sprints the CGSS significantly increased step length (*P* = 0.01, *d* = 0.91), without altering step frequency (*P* = 0.34, *d* = 0.2) and enhanced EMG activity in the *rectus femoris* muscle only (*P* = 0.01, effect size *d* = 1.24).

## Discussion

The major findings documented here are that during the final 10 of the 30 repeated sprints wearing the CGSS improved performance, lowered the rating of perceived exertion by the upper leg muscles, increased step length without altering step frequency, and enhanced muscle activation. The hip-flexion angle was reduced by this garment during all of the sprints. None of the cardio-respiratory, metabolic and hemodynamic variables monitored were affected.

Earlier research has demonstrated that performance during short bursts of explosive high-intensity effort is improved by application of compression [28,36,37], in particular (as revealed by the effect size calculation in a recent systematic review) in the case of repeated sprinting and jumping [6]. In contrast, compression was reported not to exert any positive effect on repeated 20-m sprint times during a simulated netball game [38], although the pattern of intensity and rest in this particular study was unfortunately not made clear. Moreover, performance of intermittent 15-m sprints during a simulated field hockey game was unaffected by the application of compression clothing [39], but since there were long active rest periods between these sprints, this protocol may not have induced significant muscular fatigue. In addition, compression clothing did not influence the performance of rugby players during 10 20-m sprints run at the rate of one per minute [40].

To induce extensive muscular fatigue and perceived exertion, 30 30-m sprints were employed here. Application of the CGSS did not affect performance during the early stage of this protocol, only during the final 10 sprints. A recent commentary on repeated sprinting asking for future evaluation of multiple sessions of sprinting also mentioned that pacing becomes more likely as the number of sprints is increased [41]. We cannot excluded the possibility that our participants utilized some pacing strategy.

At the same time, it has been demonstrated that the reproducibility of performance is influenced profoundly by knowing and thereby being able to anticipate the number of sprints [42], as well as by the experience of the athletes involved [43]. For these reasons, we only included subjects whose training involved repeated sprinting. Moreover, all participants knew the number of sprints to be performed beforehand, and received no feedback concerning performance at any time in order to promote equal motivation under both conditions. Therefore, we conclude that the differences in running performance observed were due to the clothing.

In sub-study 1 the CGSS did not significantly alter any of the physiological variables monitored, although the average rating of perceived exertion for the upper leg muscles was reduced during the final 10 of the 30 sprints. For patients with dysfunctional venous valves, compression garments improve local and central hemodynamics [44,45]. Application of pressure to the skin leads to redistribution of blood from the superficial to the deeper venous system, thereby improving muscle pumping and venous return [7,8]. It has been proposed that such enhanced venous return increases the end-diastolic filling and stroke volume of the heart, thereby reducing the heart rate during exercise at any level of intensity [46]. Since our present findings reveal no change in heart rate when wearing CGSS, it is questionable whether such effects observed in patients with dysfunctional venous valves will occur in healthy athletes.

Although compression clothing improves regional blood flow and thereby oxygen availability and utilization in patients suffering from venous insufficiency [47], analogous studies on well-trained endurance athletes are inconclusive. During high-intensity running, compression has been reported to enhance the blood volume, but lower oxygenation of the *vastus lateralis* muscle [9]. In contrast, others have observed no alteration in local blood volume, but elevated tissue oxygenation when wearing compression clothing during intermittent high-intensity running [10]. In addition, positron-emission tomography has recently revealed that blood flow is actually reduced after high-intensity effort wearing compression clothing [24].

The present investigation revealed no change in oxygen uptake with the CGSS, in agreement with previous findings during high-intensity endurance exercise [9,48-50]. During moderate intensity running oxygen uptake was reduced by compression clothing, which is associated with reduced oscillation of the muscle due to the mechanical support provided by the garment [13]. In general, vibration of a muscle increases its activity [51], as well as cardio-respiratory and metabolic demands [52,53]. Since compression clothing attenuates vibration of the muscle belly upon the impact of each step [28], it has been proposed that such clothing also reduces oxygen uptake at a given velocity of running [13]. Indeed, these investigators could detect such a reduction during submaximal (12 km∙h^-1^), but not higher-intensity running. Since the CGSS caused no alternations in the oxygenation index, blood volume or oxygen uptake here, mechanisms other than altered cardio-respiratory and hemodynamic function are likely to explain the improved performance we observed during repeated sprints.

The CGSS significantly lowered our subjects’ rating of perceived exertion by the upper leg muscles, which are key contributors to sprint performance [54]. In a similar manner, earlier research has shown that compression clothing lowers perceived exertion, especially in those suffering from severe muscular pain and discomfort [55-58]. However, with our experimental protocol this lower rating of perceived exertion might be a placebo effect. Perceived comfort and exertion exert an important influence on the athlete’s performance, irrespective of any physiological and/or biomechanical benefits [37,59]. However, from a practical point of view, “blinding” of our subjects was impossible, since the compression and/or the adhesive silicone stripes are easily perceived. To minimize the placebo effect no detailed information concerning the CGSS or control tights was provided.

As mentioned above, CGSS significantly reduced the hip flexion angle and increased step length, without altering step frequency. Since running velocity is the product of step length times step frequency, any increase in either will improve performance. In general, step length correlates negatively to step frequency [60], but in elite athletes step length appears to exert a larger impact on sprint performance [61]. Moreover, in well-trained, but non-elite athletes (including those involving in track-and-field and team sports), sprint velocity is correlated to step length [60]. Such findings are consistent with our current observation that the CGSS improved performance by increasing step length without changing step frequency.

Mechanoreceptors in the muscle tissue, skin, ligaments and joint capsules provide continuous feedback concerning the movement of these segments [62]. Both compression garment and sports taping have been reported to stimulate these mechanoreceptors, thereby elevating the input to neuromuscular pathways, reflex activation, and sensory feedback [18,19,63-65] and improving proprioception [66,67] and power production [15,16]. Taping alters the electromyographic activity of the *quadriceps femoris* muscle by enhancing the rate of firing and/or recruitment of motor units [15]. However, we detected no significant changes in muscle activation with the CGSS in most of the assessed muscle groups, in line with other findings [68].

Although activation during the final 10 sprints of the protocol was increased significantly with the CGSS in the *rectus femoris* muscle only, large effect sizes concerning better maintenance of muscle activation were evident for other thigh muscles as well. Such a meaningful effect, along with a likely increase in the iEMG, was particularly apparent for the *gluteus maximus* muscles (*P* = 0.13, *d* = 0.84). Power production by the hip extensors exerts a key impact on performance during the late stance phase of sprinting [69-71]. Along with the increased iEMG of the *rectus femoris* muscle, elevation in muscle activation during the late stance phase of each step due to the CGSS might have contributed here to the longer step length and improved performance of repeated sprints.

Acceleration during the initial 10 m of a sprint is characterized by leaning forward and relies primarily on force and power production by the leg extensors [72,73]. In later phases of the sprint with the body upright, running technique, including rapid touchdown, low braking forces, high reverse acceleration of the leg during the stance phase, and rapid forward movement of the leg during the swing phase, determines sprint performance [73-75]. In this later phase, enhanced sensory feedback resulting from the CGSS may have altered proprioception and running technique, explaining the improvement in performance, whereas in earlier studies compression clothing did not enhance performance during repeated sprints over shorter distances (15-m and 20-m) [38-40].

### Limitations of the study

All of the trials here were conducted when the subjects were not menstruating. In order to avoid serious interference with their training and competition schedules, they were not required to be in the exact same phase of the menstrual cycle. While early research indicated variations in exercise performance over the course of the menstrual cycle [76], a later review concludes that performance and resistance to fatigue during strength-specific exercise tasks are constant throughout the cycle [77]. A recent report on female athletes performing repeated sprints in three phases of the menstrual cycle found no effect on power production, recovery nor any of the metabolic parameters monitored due to the varying concentrations of estrogen and progesterone [78]. Therefore, we conclude that differences in running performance observed here were not due to hormonal variations.

## Conclusions

In both of our current sub-studies the CGSS improved performance in the final 10 of the 30 30-m repeated sprints. While physiological parameters remained unaffected, perceived fatigue was lowered and running technique altered by wearing this garment. Our findings indicate that the improved performance may be due more to altered sprint mechanics than to previously proposed physiological mechanisms, such as alterations in local and/or central hemodynamics or oxygen availability. Future research should be designed to clarify the potential benefits of such a compression garment with adhesive silicone stripes during repeated sprints separated by shorter intervals of rest, where performance relies more heavily on aerobic energy production and rapid recovery.

## Competing interests

This investigation was supported financially by the Swedish National Centre of Research in Sports (CIF) as well as by PUMA SE (Herzogenaurach, Germany) and own institutional resources. Neither CIF nor PUMA SE were involved in development of the study design, data collection and analysis, or preparation of the manuscript. There are no other potential conflicts of interest related to this article.

## Authors’ contributions

Conception and design of the experiments: DPB, FG, BS. Performance of the experiments: DPB, BS. Analyses of the data: DPB, FG, BS. Provision of reagents/materials/analytical tools: DPB, HCH, FG, BS. Preparation of the manuscript: DPB, HCH, FG, BS. All authors read and approved the final manuscript.

## Pre-publication history

The pre-publication history for this paper can be accessed here:

http://www.biomedcentral.com/2052-1847/6/21/prepub
